# Effect of challenge of pigs previously immunised with inactivated vaccines containing homologous and heterologous *Mycoplasma hyopneumoniae *strains

**DOI:** 10.1186/1746-6148-8-2

**Published:** 2012-01-06

**Authors:** Iris Villarreal, Katleen Vranckx, Dries Calus, Frank Pasmans, Freddy Haesebrouck, Dominiek Maes

**Affiliations:** 1 Faculty of Veterinary Medicine, Ghent University, Salisburylaan 133, B-9820 Merelbeke, Belgium

## Abstract

**Background:**

*Mycoplasma hyopneumoniae *is the primary cause of enzootic pneumonia in pigs. Although vaccination is an important control tool, the results observed under field conditions are variable. This may be due to antigenic differences between the strains circulating in pig herds and the vaccine strain. This study compared the protective efficacy of four bacterins against challenge infection with a highly virulent field strain of *M. hyopneumoniae*.

Seventy eight, one-week old piglets were randomly assigned to five treatment groups (A, B, C, D, E), 14 piglets each, and a negative control group (F) consisting of 8 piglets. All pigs were injected at 1 (D7) and 4 weeks of age (D28), with 2 ml of either a placebo or a bacterin based on selected *M. hyopneumoniae *strains, namely A (F7.2C), B (F20.1L), C (B2V1W20 1A-F), D (J strain), E (placebo; positive control), F (placebo; negative control). At D56, all pigs except those of group F were challenged intratracheally with 7 ml culture medium containing 10^7 ^CCU/ml of *M. hyopneumoniae *strain F7.2C. All pigs were euthanized and necropsied at D84. The severity of coughing and pneumonia lesions were the main parameters. Immunofluorescence (IF) testing, nested PCR testing of bronchoalveolar lavage (BAL) fluid and serology for *M. hyopneumoniae *were also performed.

**Results:**

The different bacterins only slightly improved clinical symptoms (average 0.38 in vaccinated groups vs. 0.45 in group E) and histopathological lung lesions (average 3.20 in vaccinated groups vs. 3.45 in group E), but did not improve macroscopic lung lesions (score 4.30 vs. 4.03 in group E). None of the vaccines was significantly and/or consistently better or worse than the other ones. All bacterins evoked a serological response in the vaccinated animals. All pigs, except those from group F, were positive with nPCR in BAL fluid at D84.

**Conclusion:**

The bacterins did not induce a clear overall protection against challenge infection, and there were no significant differences in protective efficacy between bacterins containing homologous and heterologous *M. hyopneumoniae *strains. Further research is necessary to better characterize the antigens involved in protection and to elucidate the protective immunity responses following *M. hyopneumoniae *vaccination and/or infection.

## Background

*Mycoplasma hyopneumoniae *is the primary cause of enzootic pneumonia in pigs, a disease causing major economic losses to the pig industry worldwide due to reduced performance and increased medication [1,2]. Control of enzootic pneumonia can be accomplished by optimizing herd management practices and housing conditions, and by using antimicrobial medication and/or vaccination [2-4].

Vaccination with the commercially available vaccines is frequently practiced worldwide. Most vaccines are adjuvanted bacterins, and they generally provide good results in terms of improving clinical symptoms and lung lesions, and in decreasing performance losses and antimicrobial medication due to *M. hyopneumoniae *infections. However, the effects of vaccination may vary from herd to herd [2], and also under experimental conditions, it has been shown that the effects of vaccination differ depending on the challenge strain used to inoculate the animals [5]. The variable results observed under field conditions may be due, among other factors, to antigenic differences between the strains circulating in pig herds and the vaccine strain. Previous studies have demonstrated that *M. hyopneumoniae *strains circulating in pig herds display considerable variation at genomic [6] and protein level [7], and also in terms of virulence, major differences between strains have been demonstrated [8,9]. The current commercial vaccines used to control enzootic pneumonia are mostly based on the J-strain, which was originally isolated from a pig herd in the UK (Goodwin and Whittlestone, 1963). It can therefore be questioned whether this strain has similar characteristics as the *M. hyopneumoniae *strains circulating in pig herds in other parts of the world [6,10].

The aim of this study was to compare the protective efficacy of four bacterins against challenge infection with a highly virulent field strain of *M. hyopneumoniae*. One of the bacterins was based on the challenge strain, two were based on other *M. hyopneumoniae *field strains and one contained the J strain.

## Methods

### Animals and experimental design

Seventy eight cross-bred pigs from a herd free of *M. hyopneumoniae *were included in the study. The herd was free from *M. hyopneumoniae *for more than 10 years based on regular monitoring of clinical symptoms, pneumonia lesions at slaughter, and serological testing for presence of antibodies against *M. hyopneumoniae*. The piglets were weaned at one week of age, and transported to the experimental facilities. They were randomly allocated to five different treatment groups (A, B, C, D, E) of 14 piglets each, and a negative control group (F) of 8 piglets. Every group was housed in a different room and each room had an own ventilation system to avoid transmission of infection from one group to another.

Pigs were vaccinated twice intramuscularly with 2 ml of either a placebo consisting of phosphate buffered saline solution (groups E and F) or a bacterin (groups A-D). The first time, they were vaccinated one day after transport to the experimental facilities (D7) and again three weeks later (D28). Vaccines A, B and C were based on field strains: A on F7.2C, B on F20.1L, and C on B2V1W20 1A-F. Previous studies revealed that these strains are genetically and antigenically different [7,11]. Vaccine D was based on the J strain (Mypravac suis^®^, laboratorios HIPRA).

Four weeks after the second vaccination (D56), all pigs of groups A-E were challenged intratracheally with 7 ml of culture medium containing 10^7 ^CCU/ml of *M. hyopneumoniae *strain F7.2C. Therefore, the pigs were anaesthetized with 0.22 ml/kg of a mixture of Xyl-M^® ^2% (Intervet) and Zoletil 100^® ^(Virbac). The negative control group F was inoculated intratracheally with 7 ml of *M. hyopneumoniae *culture medium. All pigs were euthanized and necropsied 4 weeks after challenge infection (D84). Therefore, deep anaesthesia was applied by administrating intramuscularly 0.3 ml/kg of a mixture of XylM^® ^2% and Zoletil 100^®^, followed by exsanguination. The experimental design and the different groups are presented in Table 1.

**Table 1:** Different groups and experimental design.

Group	Number of pigs	Strain used in bacterin	Challenge infection at D56	Necropsy at D84
**A**	14	F7.2C	+	+
**B**	14	F20.1L	+	+
**C**	14	B2V1W20 1A-F	+	+
**D**	14	J strain	+	+
**E**	14	placebo**^a^**	+	+
**F**	8	placebo **^a^**	- **^b^**	+

Legend: This table shows efficacy of different *M. hyopneumoniae *bacterins against challenge infection with a highly virulent *M. hyopneumoniae *strain. Pigs in each group were injected intramuscularly at D7 and D28 with the respective bacterins or a placebo solution. Euthanasia of all pigs was performed on D84.

a Phosphate buffered saline solution was used as placebo for the vaccination

b *M. hyopneumoniae *culture medium was used as placebo for the challenge infection

c Pigs were intratracheally inoculated with 7 ml of 10^7 ^CCU/ml *M. hyopneumoniae *strain F7.2C. at D56

The study was conducted after approval of the ethical committee for conducting animal experiments within CEYC (approval number EID-2009-031).

### Preparation of the bacterins

The selected *M. hyopneumoniae *strains for producing bacterins A, B and C, together with the J strain, were cultured during three weeks in modified Friis' medium [12] at 37°C in an atmosphere containing 5% CO2. The Mycoplasmas were inactivated with binary ethylamine to obtain a whole-cell bacterin in an aqueous adjuvant suspension. All bacterins were adjusted to the same *M. hyopneumoniae *cell concentration namely 7.7 log_10 _CCU/ml, before inactivation.

### Parameters of comparison and sampling of pigs

#### Clinical parameters

After intratracheal inoculation and until the end of the study, piglets were observed daily for a minimum of 15 minutes. The severity of coughing was assessed using a respiratory disease score (RDS) as described by Halbur *et al. *[13]. Body condition, appetite, presence of dyspnea and tachypnea were also recorded. The RDS could range from 0 to 6: 0 (no coughing), 1 (mild coughing after an encouraged move), 2 (mild coughing in rest), 3 (moderate coughing after encouraged move), 4 (moderate coughing in rest), 5 (severe coughing after encouraged move), 6 (severe coughing in rest). The daily RDS values were summed and averaged on D84.

#### Macroscopic lung lesions, histopathology and immunofluorescence (IF) testing for *M. hyopneumoniae*

After necropsy, the lungs were removed to score the macroscopic pneumonia lesions according to Hannan *et al. *[14], with values ranging from 0 (no lesions) to 35 (entire lung affected). Samples from the right apical, cardial and diaphragmatic lung lobe of each pig were collected for histopathological examination and IF testing. If lung lesions were present, tissue samples for IF testing were collected from the edge of a lesion.

For histopathological examination, the lung tissue samples were fixed in 10% neutral formalin, processed and embedded in paraffin. After hematoxylin and eosin staining, samples were scored using light microscopy. Scores could range from 0 to 5, according to the degree of peribronchiolar and perivascular lymphohistiocytic infiltration, as well as cuffing formation [15,16]. Scores 1 (limited cellular infiltrates- macrophages and lymphocyte- around bronchioles; with airways and alveolar spaces free of cellular exudates) and 2 (moderate infiltrates with mild diffuse cellular exudates into airways) were classified as lesions not related to *Mycoplasma *infections. Scores 3, 4, 5 were considered to be associated with *Mycoplasma *infection (mild, moderate or severe- broncho-interstitial (cuffing) pneumonia, surrounding bronchioles but extending to the interstitium, with lymphofollicular infiltration and mixed inflammatory cell exudates). The average histopathological lung score per group was calculated.

A direct IF assay [17] was performed to assess the load of *M. hyopneumoniae *organisms in a semi-quantitative way. The scores could range ranged from 0 to 3: 0 (no IF), 1 (limited IF), 2 (moderate IF), and 3 (intense IF) [8].

#### Nasal swabs, bronchoalveolar lavage fluid, and nPCR

Nasal swabs were collected from all animals at D7, D28, D56, D70 and just before euthanasia on D84. At necropsy on D84, 20 ml of PBS were slowly infused in the left lung and aspirated back into the syringe. The recovered bronchoalveolar lavage (BAL) fluid was aliquoted, immediately cooled at 4°C, and subsequently stored at -70°C until analysis.

DNA was extracted from the nasal swabs and BAL fluid with the QIAGEN protocol for purification of total DNA from animal blood and tissues (QIAGEN, DNeasy Blood & Tissue Kit, Belgium). For detection of *M. hyopneumoniae *DNA, a nPCR-test based on a modified protocol described by Stärk *et al. *[18] was performed [16]. ].

#### Serology

Blood samples were taken from all pigs at D7, D28, D56, D70 and D84 and tested for the presence of antibodies against *M. hyopneumoniae *using the DAKO^® ^Mhyo ELISA (Oxoid Limited, Hampshire, UK). Sera with inhibition percentage (IP) > 50% were considered positive, IP values < 50% were considered negative. Intermediate IP values were considered doubtful and classified as negative for the data analysis.

#### DWG and FCR

Pigs were individually weighed on D7, D28, D56, D70 and D84. The daily weight gain (DWG) and feed conversion ratio (FCR) were calculated for all groups. The DWG (g/day) for different periods during the study was estimated from the difference between the starting and final weight at a period, divided by the number of days lived by the pigs during that period. The FCR was calculated as the amount of feed consumed divided by the mean increase in live weight during each period.

#### Bacteriology

A sample of BAL fluid per pig in each group was inoculated on Columbia agar supplemented with 5% sheep blood (Oxoid, Hampshire, UK) with a *Staphylococcus pseudintermedius *streak for support of *Actinobacillus *and *Haemophilus *sp. growth, and on Columbia CNA agar with 5% sheep blood (Oxoid, Hampshire, UK). As described by Quinn *et al. *[19], plates were incubated in a 5% CO2-enriched environment at 37°C for 48 h for identification of respiratory bacteria in the lungs.

### Statistical analysis

The RDS scores, macroscopic, histopathological lung lesions, IF scores, serology (IP values) and DWG in the different groups were statistically analyzed using one-way analysis of variance (ANOVA). For each parameter, pair-wise comparisons between groups were obtained using Bonferroni's test. The proportion of serologically positive pigs, and pigs positive by nested PCR on nasal swabs and BALF at different time points (D7, D28, D56, D70 and D84) were analyzed using logistic regression. Group F was excluded from the statistical analysis as this group only served as a sentinel group.

Statistical results were considered significant when P ≤ 0.05 (two-sided test). The statistical package SPSS 17.0 for Windows (SPSS 17.0, SPSS Inc., IL, USA) was used for the analysis.

## Results

### Clinical parameters

Throughout the total duration of the trial, coughing was absent in group F. The onset of coughing occurred first in groups C and E at 7-8 DPI, followed by group B, D and A at 11, 13 and 14 DPI, respectively. The RDS recorded for groups A, B, C, D, E and F were 0.29, 0.50, 0.53, 0.18, 0.61 and 0, respectively (Table 2). No significant differences were observed between groups A, B, C and E (P > 0.05). The RDS was significantly lower in group D than in group E (P < 0.05).

**Table 2:** Results (mean ± SD) of the different parameters.

Parameter	Treatment group					
	**A**	**B**	**C**	**D**	**E**	**F**

RDS	0.29 ± 0.09^a,b,c^	0.50 ± 0.21^a,b^	0.53 ± 0.25^a,b^	0.18 ± 0.14^,a,c^	0.61 ± 0.32^b^	0.00 ± 0.00^c^
Macroscopic lung lesions	6.86 ± 4.54^a^	2.59 ± 1.39^b,c^	3.33 ± 0.97^b,c^	4.59 ± 2.82^a,b,c^	4.06 ± 2.45^a,b^	0.23 ± 0.35^c^
Histopathological lung lesions	3.49 ± 0.67^a^	3.48 ± 0.65^a^	2.89 ± 0.74^a^	2.93 ± 0.76^a^	3.45 ± 0.73^a^	1.29 ± 0.30^a^
IF	1.63 ± 0.97^a,b^	0.91 ± 0.86^a,c^	1.27 ± 0.21^a,b,c^	1.77 ± 0.70^a,b^	2.23 ± 1.05^b^	0.14 ± 0.38^c^

Respiratory disease score (RDS), macroscopic and histopathological lung lesions, and immunofluorescence (IF) scores for *M. hyopneumoniae *in the lung tissue in the different groups (Groups A-E: n = 14 pigs per group; group F: n = 8 pigs)

Group A (F7.2C), B (F20.1L), C (B2V1W20 1A-F), D (J strain), E (positive control; non-vaccinated, challenge infected), F (negative control; non-vaccinated, non-challenged).

Within a row, different superscript letters correspond to significant differences between the groups (P < 0.05)

### Macroscopic, histopathological lung lesions and IF testing for *M. hyopneumoniae*

The macroscopic lung lesion scores in groups A, B, C, D, E and F were 6.86, 2.59, 3.33, 4.59, 4.06 and 0.23, respectively. Only two pigs had very mild lung lesions in group F. Group A had significantly higher scores than group B and C (P < 0.05). There were no significant differences between groups B, C, D and E (Table 1).

The scores for the histopathological lung lesions in groups A, B, C, D, and E were 3.49, 3.48, 2.89, 2.93 and 3.45, respectively (P > 0.05) (Table 1). The pigs in group F did not show Mycoplasma-like histopathological lesions. The average score (1.29) in group F was significantly lower than the scores of the other groups (P < 0.05).

All pigs, except for those in group F, were positive for IF staining. The highest IF score was recorded in group E (2.23), the lowest in group B (0.91) (P < 0.01). The scores in groups A, C and D were 1.63, 1.27 and 1.77, respectively (P > 0.05). One pig from group F had a positive IF testing in one of the 3 sampled lobes.

### nPCR testing on nasal swabs and BAL fluid

Presence of *M. hyopneumoniae *was confirmed in all infected groups. The results of the nPCR on nasal swabs at different points and the nPCR testing on BAL fluid at D84 are presented in Table 3. All animals in group F tested negative at all time-points. The majority of the animals in the infected groups (A-E) tested positive in nasal swabs at D70 and D84. All of them were positive in the BAL fluid at D84.

**Table 3:** Number (%) of positive pigs by nested PCR.

Age of pigs	Treatment group					
	A	B	C	D	E	F
D7	0/14	0/14	0/14	0/14	0/14	0/14

D28	0/11	0/12	0/9	0/11	0/11	0/7
D56	0/11	0/12	0/8	0/11	0/11	0/7
D70	6/10^a,b^	6/11^a,b^	7/7^a^	8/10^a^	7/10^a^	0/7
D84	7/10	7/11	3/5	5/10	7/10	0/7
D84 (BALF)	10/10	10/10	5/5	10/10	10/10	0/7

Legend: nested PCR was carried on nasal swabs (D7, D28, D56, D70, D84) and bronchoalveolar lavage fluid (BALF) (D84) throughout the trial (Groups A-E: n = 14 pigs per group; group F: n = 8 pigs)

Group A (F7.2C), B (F20.1L), C (B2V1W20 1A-F), D (J strain), E (positive control; non-vaccinated, challenge infected), F (negative control; non-vaccinated, non-challenged).

Within a row, different superscript letters correspond to significant differences between the groups (P < 0.05). Group F was not included in the statistical analysis.

### Serology

The percentage of seropositive pigs in each group and the average IP values are presented in Table 4. Piglets in group F remained negative throughout the entire study. All pigs from groups A-E tested serologically negative at D7. The first seropositive pigs were identified at D28 *i.e*. 3 weeks after the first vaccination, in all vaccinated groups except for group A. The pigs of group E remained serologically negative until 2 weeks after challenge infection (D70). At that time (D70), 60% of the pigs were serologically positive in group E, whereas all pigs were serologically positive in the vaccinated groups (A-D). At D84, all pigs from groups A-E were serologically positive.

**Table 4:** Percentage of seropositive pigs and inhibition percentage (IP) in each group.

Age of pigs	Treatment group					
	**A**	**B**	**C**	**D**	**E**	**F**

D7	0	0	0	0	0	0
D28	0^A,C ^(35.5)^a^	17^A ^(36.2)^a^	89^B ^(54.9)^b^	9^A ^(32.7)^a^	0^C ^(8.34)^c^	0
D56	100^A ^(76.4)^a^	100^A ^(75.5)^a^	100^A ^(89.3)^b^	100^A ^(73.4)^a^	0^B ^(14.1)^c^	0
D70	100^A ^(92.6)^a^	100^A ^(92.7)^a^	100^A ^(93.5)^a^	100^A ^(93.6)^a^	60^B ^(63.6)^b^	0
D84	100 (96.4)^a^	100 (96.3)^a^	100 (96.7)^a^	100 (96.4)^a^	100 (84.9)^b^	0

Legend: Serology was performed using DAKO^® ^M.hyo ELISA (Oxoid Limited, Hampshire, UK) for *Mycoplasma hyopneumoniae *in each group (Groups A-E: n = 14 pigs per group; group F: n = 8 pigs)

Group A (F7.2C), B (F20.1L), C (B2V1W20 1A-F), D (J strain), E (positive control; non-vaccinated, challenge infected), F (negative control; non-vaccinated, non-challenged).

Sera with IP value > 50% was considered positive, IP values < 50% were considered negative. Intermediate IP values were considered doubtful and classified as negative.

Within a row, different superscript letters correspond to significant differences between the groups (P < 0.05): capital letters for the proportion of seropositive pigs, lowercase letters for the IP values. Group F was not included in the statistical analysis.

### Bacteriology

No relevant bacterial growth was observed at 2, 4, nor 7 days after plating the BALF samples on Columbia blood agar and CNA plates.

### Performance

The results of DWG and FCR are presented in Table 5. Significant differences between groups for DWG were only observed during the specific periods of the study (D28-D56, D56-D70, D70-D84), but there were no significant differences for the entire study period (D28-D84). The overall DWG was highest in groups A, D and F. These groups together with group B also had the lowest FCR.

**Table 5:** Daily weight gain (DWG) (g/day) (mean ± SD) and feed conversion rate (FCR) for each group (A-F).

Period	Parameter	Treatment group					
		**A**	**B**	**C**	**D**	**E**	**F**

D7-28	DWG	154.5 (72.2)	155.9 (64.4)	146.9 (64.6)	151.3 (49.6)	158.7 (42.0)	180.5 (49.0)
D28-56	DWG	415.5 (92.2)	353.9 (110.9)	315.6^a ^(92.5)	359.2 (76.4)	338.9 (87.8)	469.1^b ^(118.1)
	FCR	1.38	1.18	1.30	1.42	1.50	1.31
D56-70	DWG	449.8^a,b ^(252.5)	425.5^a,b ^(213.3)	293.8^a ^(157.9)	632.9^b ^(143.0)	506.4^a,b ^(221.7)	700.7^b ^(222.0)
	FCR	2.00	2.07	2.37	1.50	1.90	1.56
D70-84	DWG	697.1 (61.4)	679.9 (113.5)	505.7 (42.0)	552.8^a,b^(144.0)	653.2 (97.2)	654.9^b ^(169.4)
	FCR	1.77	1.72	2.13	1.92	1.87	1.78
D28-84	DWG	491.7 (84.7)	452.7 (146.4)	413.3 (63.2)	486.7 (76.1)	460.3 (101.6)	486.7 (106.9)
	FCR	1.74	1.72	2.04	1.61	1.80	1.52

Legend: Groups A-E: n = 14 pigs per group; group F: n = 8 pigs.

Group A (F7.2C), B (F20.1L), C (B2V1W20 1A-F), D (J strain), E (positive control; non-vaccinated, challenge infected), F (negative control; non-vaccinated, non-challenged).

No FCR was measured during D7-28

Within a row, different superscripts correspond to significant differences between groups (P < 0.05). As FCR was measured at group level, no statistical analysis was done to compare the groups.

The number of pigs that died in the different groups were 4, 3, 9, 4, 4 and 1 in groups A, B, C, D, E and F, respectively. The majority of them died during the pre-challenge phase due to *E. coli *diarrhea. One animal died during anesthesia, and in two other animals (group C), a non-serotypable *Streptococcus suis *strain was isolated from the lung and from the lung and liver, respectively. Clinically diseased pigs were medicated *per os *(1 ml/piglet) with a combination of gentamicin, sulfadimidin, trimethoprim and dexamethasone (Coliprajet^®^, Hipra) for 3 consecutive days.

## Discussion

In the present study, all bacterins evoked a serological response in the vaccinated animals. However, after challenge with a highly virulent *M. hyopneumoniae *strain, a clear protection against macroscopic lung lesions was not demonstrated in these animals. This confirms a lack of correlation between serum antibodies and protection [20-22]. Studies aiming to elucidate immune mechanisms playing a role in protection against infection with this very fastidious micro-organism are urgently needed.

It is remarkable that vaccination with a bacterin based on the homologous *M. hyopneumoniae *strain did not result in an increased protection in comparison with bacterins containing other strains, including the J-strain which has been shown to be antigenically quite different from the challenge strain [7,11]. In a previous study, it was demonstrated that the effect of vaccination with a whole cell bacterin on the course of an experimental *M. hyopneumoniae *infection may vary, depending on the strain used for challenge [5]. Further studies are required to elucidate the importance of the *M. hyopneumoniae *strain included in the vaccine for induction of protection. Such studies may include vaccines with different amounts of antigen and different adjuvants.

The challenge infection was successful because in all infected pigs, lung lesions were induced and *M. hyopneumoniae *was demonstrated by nPCR testing in BAL fluid at the end of the study. The values for RDS and lung lesions in the positive control group E (non-vaccinated, infected) were similar to those found in previous experimental studies using the same challenge strain [4,5,16]. Based on nPCR testing, serology and histopathology, the negative control group F remained negative for *M. hyopneumoniae *throughout the study. This confirms that the pigs used in this study were free of *M. hyopneumoniae*. It is not clear which pathogen may have caused the very mild pneumonia lesions in two pigs of group F, and neither what may be the reason for the positive IF testing found in one of these pigs (only one of the three sampled lung lobes).

Vaccination with commercial *M. hyopneumoniae *bacterins generally provides significant improvement of clinical symptoms and lung lesions [1,2]. In the present study, clinical symptoms (average 0.38 in vaccinated groups versus 0.45 in group E) and histopathological lesions (average 3.20 in vaccinated groups versus 3.45 in group E) were lower in the vaccinated groups, but the difference was only moderate, and smaller than in previous experimental infection studies [5,16]. In addition, the macroscopic lung lesions in the vaccinated groups (4.34) were on average similar to those in the positive control group (4.03). The reasons for this are not clear. The number of *M. hyopneumoniae *organisms was kept similar in the different bacterins to avoid that concentration of *M. hyopneumoniae *cells in the different bacterins would have biased the results. It is possible that the concentration in the bacterins of the present study (7.7 log_10 _CCU/ml, before inactivation) was lower than in commercial bacterins, and that this can (partially) explain the poor or moderate efficacy. The antigen concentration in commercial bacterins is expressed in different units and therefore not directly comparable with the concentration of the present bacterins.

The bacterins in the present study were not able to prevent colonization nor to eliminate *M. hyopeumoniae *from the lung tissue, as the percentage of pigs with positive nPCR in nasal swabs and BAL fluid were the same for groups A-E. Previous studies had shown that also commercial vaccines cannot prevent colonization with *M. hyopneumoniae *and are not able to significantly reduce the transmission of the organism. All bacterins did induce serum antibodies rather quickly, as in all vaccinated groups except for group A, serum antibodies were already detected at D28 i.e. 3 weeks after the first vaccination. At the time of challenge (D56), all vaccinated pigs had detectable serum antibodies.

Performance parameters are generally included in experimental studies with *M. hyopneumoniae *[5,8] mainly because it is not that cumbersome to weigh a small number of pigs and to measure feed consumption. In general, no significant results are obtained because of the high standard deviation for these parameters and the rather small number of pigs included in experimental studies. This was also the case in the present study. The rather high number of pigs that died during the study, mainly due to *E. coli *infection before challenge, may also have influenced the performance of the pigs. Although performance parameters are very important from a financial point of view, these parameters are better investigated under practical circumstances rather than under experimental conditions.

## Conclusion

In conclusion, no significant differences in protective efficacy were observed between three newly developed bacterins based on *M. hyopneumoniae *field strains and a bacterin based on the J-strain. Further research is necessary to better characterize the antigens involved in protection and to elucidate the protective immunity responses following *M. hyopneumoniae *vaccination and/or infection.

## Authors' contributions

IV performance of the trial in the field, data analysis and writing of the manuscript, KV preparation of the inoculate and lab analysis, DC selection of strains to be used in the inoculum, FP, FH and DM development of protocol, planning of the study, and design of the clinical trial as well as review of the final manuscript, approval for publication. All authors read and approved the final manuscript.
